# Assessment of compression forces in a digitally modified short leg cast for pressure injury risk monitoring in healthy children

**DOI:** 10.3389/fped.2023.1273829

**Published:** 2024-01-18

**Authors:** Matia Iva Vrankovic, Simon Annaheim, Jana Willibald, Jan Lieber, Hubertus J. A. van Hedel, Anna-Barbara Schlüer, René M. Rossi, Andreas Meyer-Heim

**Affiliations:** ^1^ Swiss Children’s Rehab, University Children’s Hospital Zurich, University of Zurich, Affoltern am Albis, Switzerland; ^2^ Children's Research Center, University Children's Hospital Zurich, University of Zurich, Zurich, Switzerland; ^3^ Empa, Laboratory for Biomimetic Membranes and Textiles, St. Gallen, Switzerland

**Keywords:** pressure injury, modified short leg cast, compression forces, risk assessment, children, pressure sensors

## Abstract

**Introduction:**

Casting is an essential treatment for neuro-orthopedic conditions in children with cognitive, sensory, and communicational disabilities. However, a main side-effect is the development of pressure injuries resulting in additional (wound) therapies and prolongation of the hospital stay. The primary aim of our study was to investigate the potential of objective pressure measurements in casts to assess the risk for pressure injury development.

**Methods:**

Five pediatric healthy participants were included in this study. We measured the global and the local compression force at body sites prone to pressure injury development for different body positions and the transfer in-between in a cast equipped with pressure sensors. These conditions resulted in partial or full body weight loading.

**Results and discussion:**

The global maximum compression force was affected significantly by body postures with partial and full loading of the cast and during transfer. The local compression force significantly correlated with the global compression force at the heel and instep area. In conclusion, the integration of sensing technologies into casts bears a high potential for early recognition of critical conditions inside the cast and inducing preventive measures in the at-risk population.

## 1 Introduction

Pressure injuries (PIs) are localized tissue damages to the skin and underlying soft tissues, usually over bony prominences or in conjunction with medical devices (1). PI development is mainly due to intense or prolonged pressure in combination with shear forces (2). Patient characteristics that place patients at risk for PI development include reduced communication (i.e., young age, altered mental status, or developmental delay), decreased sensation (i.e., nerve blocks, myelodysplasia), and impaired mobility (i.e., spasticity and cerebral palsy or due to prosthesis, body brace, plaster cast) (3–8). Specific risk factors for PI development vary with the child's age (9). For example, neonates and infants are more prone to medical device-related PIs (10–13), whereas older children are more susceptible to PIs due to limited mobility (10, 14). For hospitalized children allocated to different pediatric units, the prevalence values across all severity levels for PI have been reported from 1.4% (15) to 26% (14, 16) and as high as 35% (17). Hospital-acquired pressure injuries have been primarily reported in pediatric intensive care units, rehabilitation units (15) and in association with medical device usage (18). Most of these PIs were NPIAP pressure injury stage 1 (non-blanchable erythema of intact skin) and 2 (partial-thickness skin loss with exposed dermis) (15, 19).

Cast immobilization is the primary treatment for many (neuro-) orthopedic conditions in children, despite many cast-related complications, including stiffness, compartment syndrome, and PIs. The incidence rate of cast-related skin complications across pediatric and adult populations has been estimated at 8.9 per 1,000 (20). It remains challenging to assess the global and local pressure impact of a cast in an objective manner to evaluate the risk for developing complications, particularly PIs, as the cast covers the risk area and, therefore, does not allow a visual clinical judgment. PIs must be considered a severe complication of cast immobilization with the potential necessity of aborting cast treatment, prolonged wound therapy or plastic surgical consultation (21). Non-removable casts place patients at risk not only for PIs but also for tissue necrosis (20), making the patient susceptible to infection (22), including osteomyelitis (3, 23). Eighty percent of the complications occur on the foot (mainly heel and ankle) and forearm (particularly elbow). The heel is the anatomic location with the most significant number of skin complications, most of which are PIs (20). Cast-related skin complications extend the patient's hospital stay, retard the rehabilitation process (24), and consequently pose a significant additional burden to the healthcare system (25). Thus, solid motivation exists to prevent pediatric PI development (26). Most PIs are preventable by adhering to evidence-based PI prevention strategies, such as educating the clinical team, regularly repositioning the patient, and nutrition consultation (27).

A systematic review explored twelve pediatric PI risk scales towards their ability to reduce PI incident in pediatric care (28). No scale revealed an outstanding performance, and it remained unclear if a PI risk assessment could reduce the PI incident in pediatric care. The authors concluded that clinical judgment might be more efficient in evaluating PI risk than applying risk scales (28). Accordingly, vigilant evaluation of patient complaints is imperative to identify an impending PI and enable revision casting, either cast windowing or cast removal (29). This necessary feedback can be reduced in the at-risk population.

Digital technologies are being developed to detect a prodromal phase of PI based on more objective screening approaches. Currently, sensing technologies enable the monitoring of local pressure impact, temperature, humidity, biomarkers in blood or urine samples, or sub-epidermal moisture, and provide several imaging modalities (30). However, these approaches have yet to be used with cast immobilization. Tuen et al. successfully measured pressure, temperature, and humidity in a short arm cast on a simulated plastic arm (31). This provided reliable input data to adjust the swathed cast's tightness to avoid complications (31).

Regarding lower extremities immobilization, a simulation model using pressure sensors placed at the first metatarsal and talar head, was created to measure cast application pressure objectively (31). Applied pressure over the measured prominences varies with the user's experience (32). Regarding the wall load of a total contact cast, the anterodistal lower leg and the posterolateral distal regions of the lower leg bear considerable load, resulting in a mechanical pressure reduction of the foot (33). Begg et al. developed a total contact cast equipped with capacitance sensors, to measure the pressure distribution in the total contact cast.

However, to our knowledge, these technological advances have yet to be routinely used in clinical practice so far. Also, no similar study could be found in a pediatric population.

In conclusion, despite the associated risk of PI development, the application of casts remains an essential therapeutic intervention for numerous neuro-orthopedic conditions. The effectiveness of specific preventive measurements, such as risk assessment scales, remains unclear, and to our knowledge, none of these technological advances has been routinely used in clinical practice so far. Furthermore, no studies investigating the application of digital technologies in casts among a pediatric population could be identified.

Therefore, the primary aim of our study was to measure and compare the global and the local compression force over body sites prone to PI development during predefined body positions with either partial or full body weight loading in a short leg cast equipped with pressure sensors in healthy children and adolescents.

The secondary aim of our study was to measure the changes in skin physiology parameters at the lateral instep area before and after the removal of the short leg cast. Data for the relative change in skin erythema, skin firmness, skin resilience, and skin elasticity were collected.

## 2 Material and methods

### 2.1 Participant recruitment

The study was conducted at the Swiss Children's Rehab in Affoltern am Albis, University Children's Hospital Zurich, Switzerland, in accordance with the Declaration of Helsinki and legal and institutional standards. Ethical approval from the cantonal ethics committee was obtained (BASEC 2020-03037). The participants and their parents gave oral consent before applying the short leg cast. The inclusion criteria for the participants were good health, normal child development, and age between 7 and 16 years. Exclusion criteria were open lesions or wounds, the usage of medication (analgesics, sedation), incontinence, orthotics or reduced mobility, abnormal body mass index (BMI, underweight (BMI < 18.5) or overweight (BMI > 25.0)) and loss of sensation or paresis.

### 2.2 Visual inspection and documentation

Before applying the short leg cast, the children were examined for peripheral sensory deficits. The CMS (circulation, motor, sensory) testing of the legs was carried out before the cast application and routinely performed during the measurements. In addition, we recorded personal data (age, weight, height, sex) and information about regular physical activity (Table 1). We measured vital parameters once before cast application (blood pressure, body temperature, oxygen saturation level, and pulse (Table 1). Furthermore, a physician inspected the child's right leg to examine and document lesions, wounds, or swelling. Pictures were taken of the whole leg, the ankle, the heel, the midfoot, and the sole. The circumference of the lower leg (10 cm below the caput fibulae), the upper ankle joint, and the midfoot were measured before and directly after the cast application to quantify potential swelling or compression. During the measurement, the child was asked to convey comfort level using a visual analog scale (VAS) (34). VAS 0 indicates no pain or discomfort. VAS 10 indicates maximum pain or discomfort. The VAS score was immediately collected after the cast application and each change in body posture. We selected the VAS score as it is a simple and reliable method of assessing pain in children as young as three years (35). A parent was present throughout the measurement. The cast could be removed at any point in case of pain or discomfort.

**Table 1:** Participant characteristics.

Participant	m/f	Age	Height [cm]	Weight [kg]	Sporting activity	Blood pressure [mmHg]	Temperature [°C]	Oxygen saturation level [%]	Pulse [bpm]	Duration of wearing the cast [min]
ID01	f	9	142	41	Hip-hop 3x/week	109/71	36.9	97	82	60
ID02	m	12	168	55	Soccer 3x/week	162/62	37.3	96	84	50
ID03	m	12	154	43	Soccer, basketball	108/61	37.3	98	90	45
ID04	f	15	153	44	Gymnastics, soccer, 3x/week	103/58	36.7	97	75	60
ID05	f	11	142	34	Floorball, gymnastics	119/68	36.6	99	96	104
ID06	f	10.5	129	26	Gymnastics, karate, horse riding	106/57	36.9	98	72	100

m, male; f, female.

#### 2.2.1 Application of the short leg cast

The same trained and experienced healthcare professional applied the short leg cast to the child's right leg. The bony prominences (lateral/ medial malleolus and instep area) were covered with an elastic foam plaster (3M™ Microfoam™ Medizinisches Schaumpflaster, 3M GmbH, Health Care, Rüschlikon, Switzerland) to ensure protection against the sensors and the cast. A tubular padding covering the whole leg was applied as the first layer of the cast (Polsterschlauch Frottée-Stretch, Tobler & Co. AG, Rehetobel, Switzerland). The pressure sensors were positioned on the padding and partially fixated with medical tape. In addition, a thin foam bandage (Tensoban Polsterschaumbinde, BSN medical GmbH, Hamburg, Germany) was used to position and fixate the sensor. Finally, a soft cast and a scotch cast (3M Schweiz GmbH, Health Care, Rüschlikon, Switzerland) surrounding the lower leg were applied according to the University Children's Hospital Zurich guidelines.

#### 2.2.2 Experimental protocol

We investigated the global compression force inside the short leg cast for 16 predefined body postures, resulting in either full or partial body weight loading of the cast. In addition, compression forces were recorded during the transfer in between body postures. Body postures with partial body weight loading of the cast included seven positions: supine, supine with elevation of the leg with cushions with heel contact, supine with elevation of the leg with splints without heel support, lateral position on the right, lateral position on the left, a sitting position with the leg extended and a sitting position with the leg bent. The transfer included seven conditions: transfer from supine position to sitting with heel support, transfer from a sitting position with heel support to a sitting position without heel support, transfer from a sitting position to the lateral side on the right, transfer to the lateral side on the left, transfer from the lateral left side to a sitting position with an extended leg, transfer to a sitting position with a bent leg, and finally transfer to standing up. The body position with full body weight loading of the cast included two positions: standing and walking. The duration of each body posture was 30 s.

### 2.3 Pressure measurement

**Figure 1:**
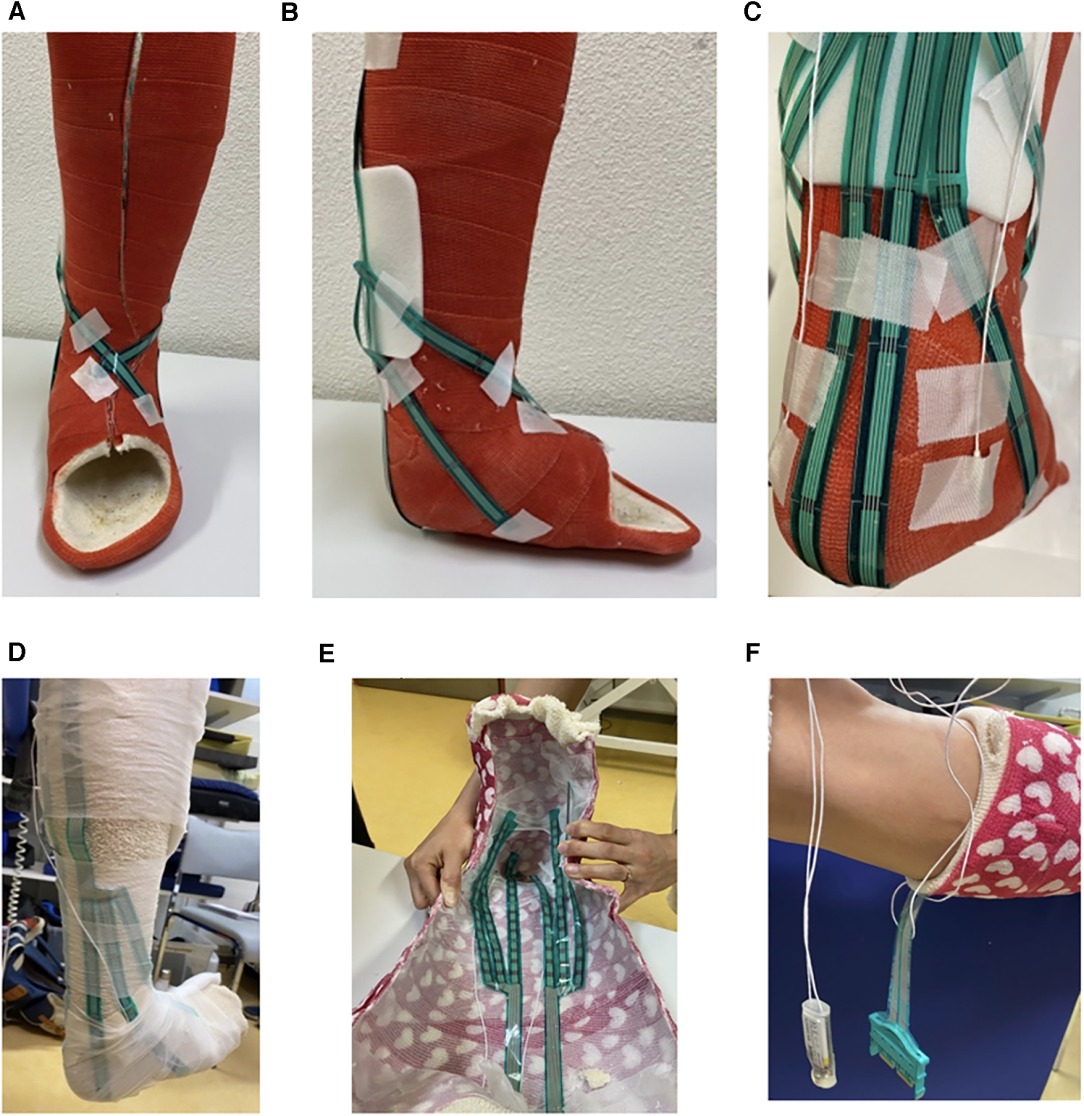
(**A–C**) Frontal, medial, and dorsal view of a prototype of the plaster cast with the placement of the sensor strands, which cover critical sites for PI development, including the instep area (**A,B**), the malleolus (**B,C**) and the heel (**C**) The sensor strands were placed to the outside of the cast in the pictures for better visualization. (**D**) Dorsal view of a participant's right leg. The pressure sensor strands are placed on the tubular padding and fixed by a thin foam bandage. (**E**) Frontal view of the location of the sensors inside the cast. (**F**) Lateral view of the placement of the leaving wires of the sensors.

**Figure 2:**
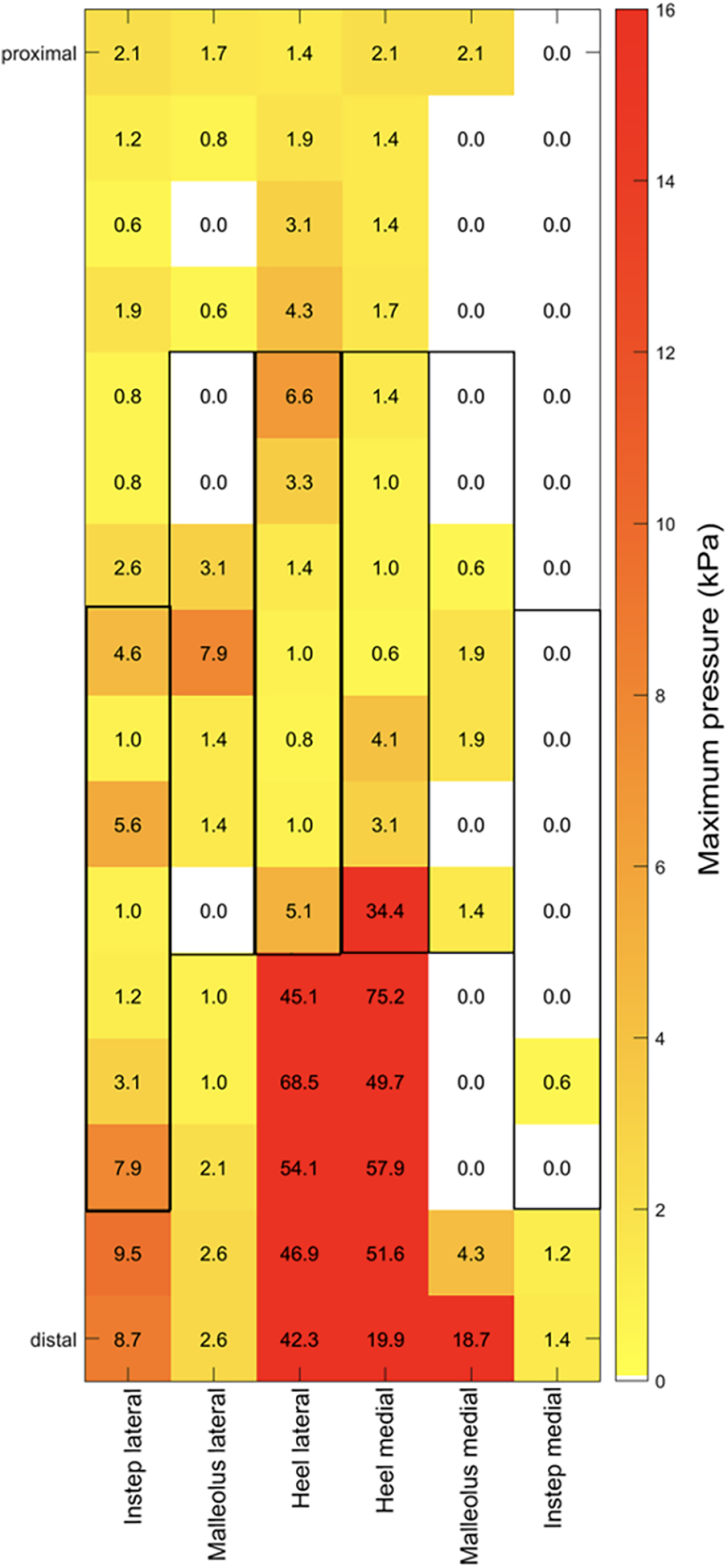
The pressure data in kPa is indicated for each sensel as recorded by the pressure mapping sensor 9,801 (Tekscan Inc., South Boston, MA, USA). Each sensor strand (indicated as lateral instep, lateral malleolus, lateral heel, medial heel, medial malleolus, medial instep) was placed over the designated body site prone to PI development. The gray boxes represent the seven sensels covering the corresponding location.

The compression force distribution inside the cast was recorded using the pressure mapping sensor 9,801 (Tekscan Inc., South Boston, MA, USA). Compression data were recorded at a 50 Hz sampling frequency for 30 s for each body posture. The pressure mapping sensor consists of six sensor strands, each including 16 pressure-sensitive areas (sensels) covering a pressure range of 34 kPa. The maximum values for global compression force includes the data from all 96 sensels inside the short leg cast. To enable an optimal alignment of the sensors covering the body sites prone to PI development, the sensor was cut along the designated lines. The individual sensor strands were placed over anatomical risk areas for developing PIs, including the medial and lateral parts of the heel, the lateral and medial instep areas and the medial and lateral malleolus (Figure 1). The maximum local compression force at the instep, the malleolus lateralis, and the heel area were calculated based on seven sensels covering the corresponding location (Figure 2).

### 2.4 Skin physiology measurement

The maximum local compression force at the instep area inside the short leg cast was compared to relative changes in skin physiology parameters. Data were collected at the lateral instep before the application and immediately after removing the short leg cast. Erythema and changes thereof were measured based on light absorption properties of the skin (Mexameter® MX18, Courage + Khazaka electronic GmbH, Cologne, Germany). Elastic properties of the intact skin such as skin firmness, skin elasticity, and skin resilience and changes thereof were quantified based on measurements of the deformation characteristics of the epidermis and stratum corneum exposed to vacuum (Cutometer®MPA580, Courage + Khazaka electronic GmbH, Cologne, Germany). Changes in skin physiology parameters were plotted against the maximum compression observed at the lateral instep.

### 2.5 Statistical analysis

For statistical analysis, IBM SPSS Statistics for Windows, Version 28.0 (IBM Corp, Armonk, NY, USA) and for data visualization, GraphPad Prism Version 9.4.1 (458), was used (GraphPad Software, LLC, San Diego, CA, USA). For all statistical analyses, α was set at 0.05.

The Shapiro-Wilk test revealed a non-normal distribution for most of the data. Therefore, data are presented as median [interquartile range (IQR)], and non-parametric tests were conducted for the statistical analyses. Differences in global and local compression forces between the three body postures (partial/ full body weight, transfer) were assessed by the Friedmann test, followed by pairwise Wilcoxon tests with a Bonferroni correction to adjust for multiple testing. We quantified the associations between the global compression forces and local compression forces and between local compression forces and changes in skin physiology parameters using Spearman's rank correlations (*ρ*). We interpreted the magnitude of the Spearman correlations according to Mukaka (2012) (36): 0–0.29 negligible, 0.30–0.49 low, 0.50–0.69, moderate, 0.70–0.89 high, and ≥0.90 very high.

## 3 Results

Six participants were included in this preliminary study (Table 1). Unfortunately, due to unreliable and defective sensor signals, the data of one participant (ID06) were excluded for further examination. Therefore, the results of the five remaining participants are presented.

### 3.1 Global maximum compression forces during predefined body postures

**Figure 3:**
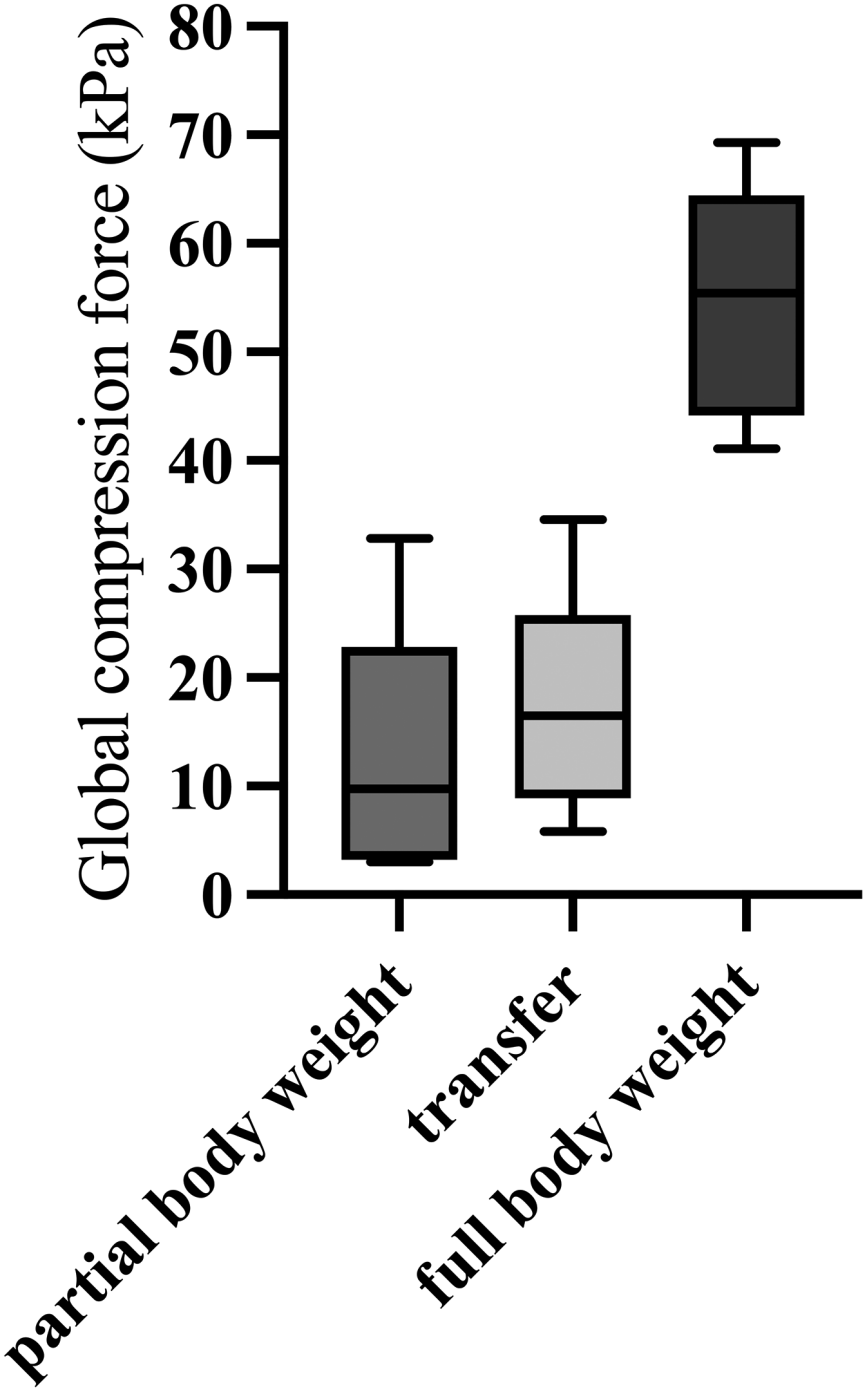
Box and whisker plots showing the global maximum compression forces during body postures with partial and full body weight and during transfer (*n* = 5). kPa, kilopascal.

The global maximum compression forces (Figure 3) differed significantly between partial and full body weight conditions and during transfer (Friedmann, *p* = 0.007). The global compression forces were significantly lower in body postures with partial body weight [9.7 kPa (IQR 3.5–12.8 kPa)] compared to the transfer condition [16.5 kPa (IQR 12.0–16.9 kPa); *p* = 0.04]. Body postures with full body weight resulted in significantly higher compression forces [55.4 kPa (IQR 47.2–59.6)] compared to partial body weight (*p* = 0.04) and transfer (*p* = 0.04).

### 3.2 Local maximum compression force during predefined body postures

**Figure 4:**
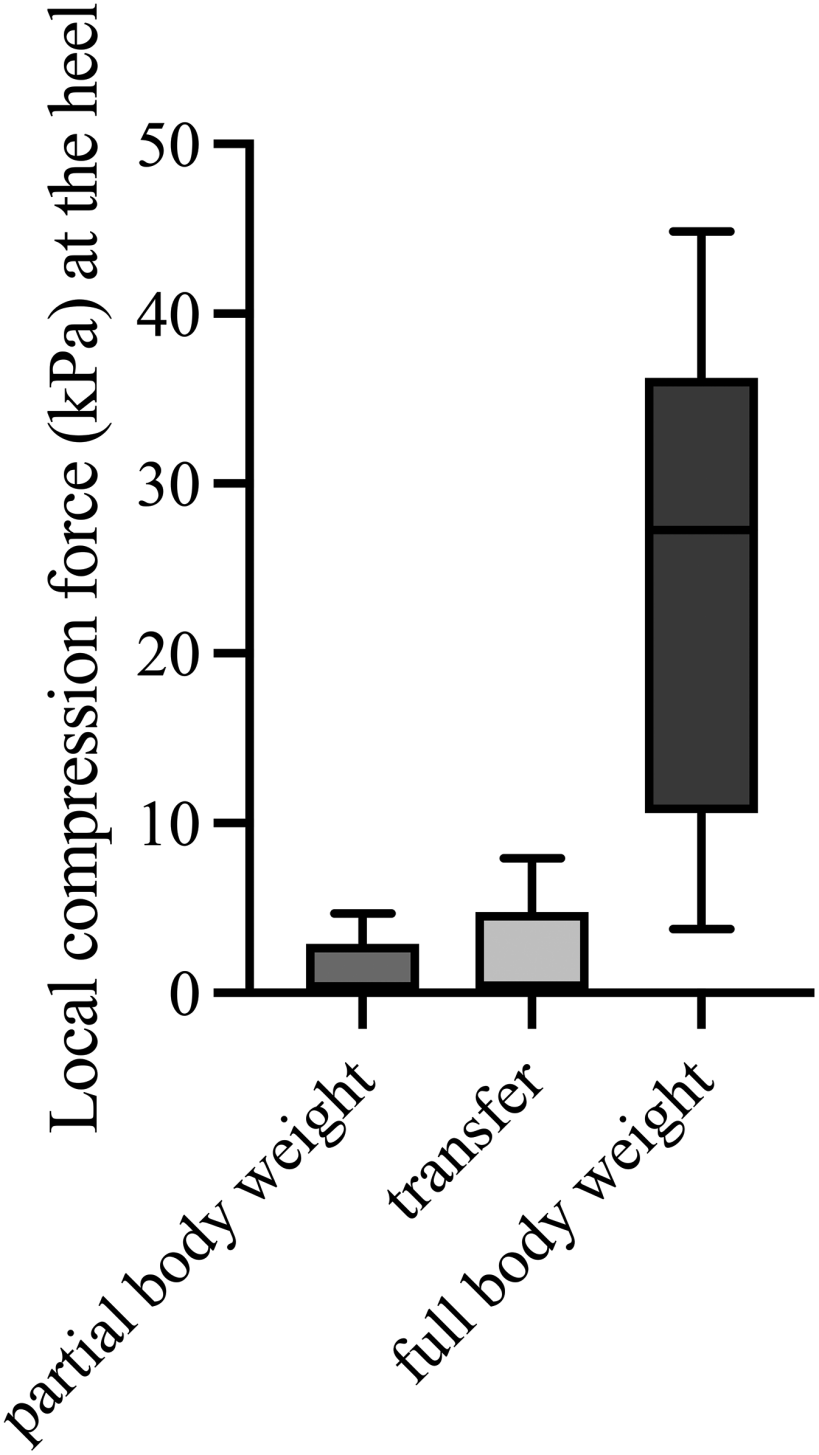
Maximum local compression forces at the heel during body postures with partial and full body weight and transfer (*n* = 5). kPa, kilopascal.

**Figure 5:**
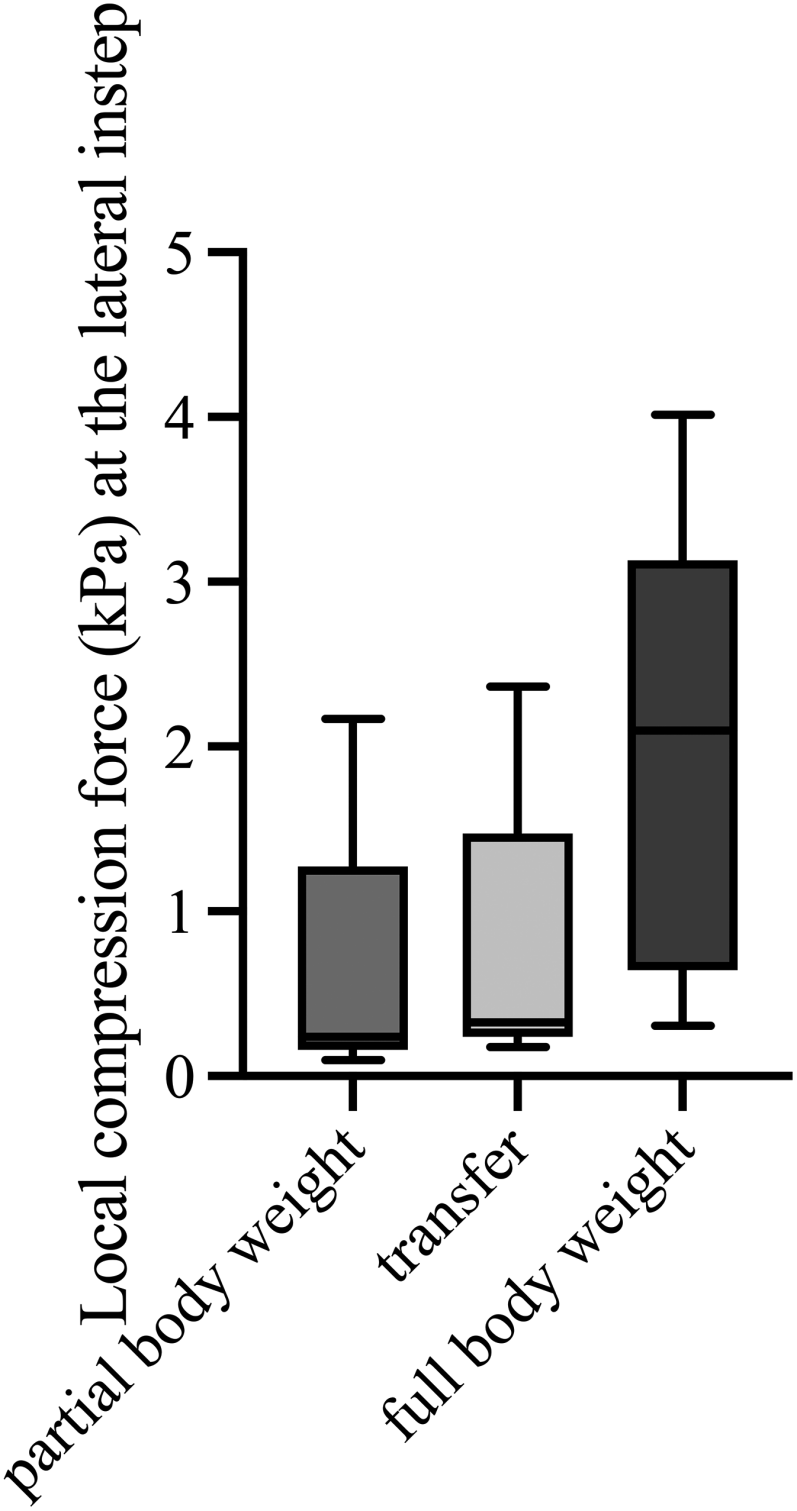
Maximum local compression forces at the lateral instep during body postures with partial and full body weight and transfer (*n* = 5). kPa, kilopascal.

**Figure 6:**
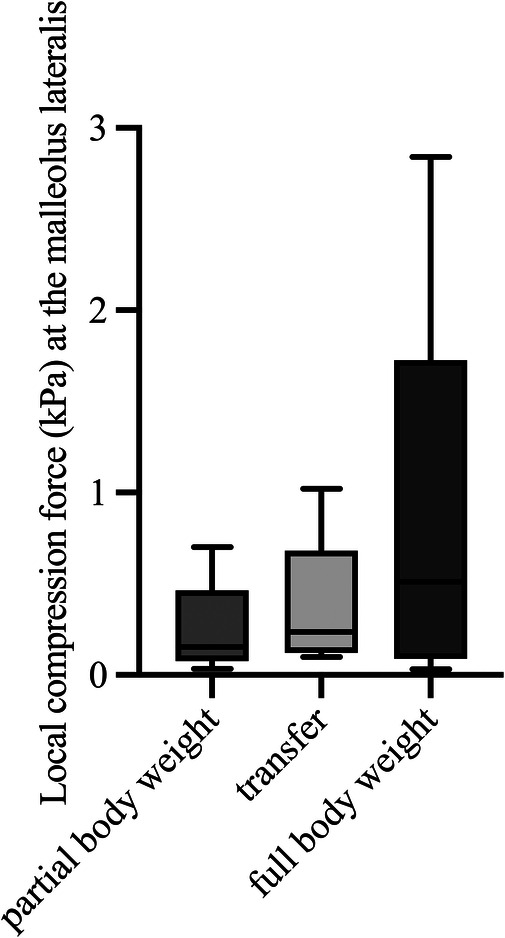
Maximum local compression forces at the malleolus lateralis during body postures with partial and full body weight and transfer (*n* = 5). kPa, kilopascal.

The local maximum compression forces at the heel (Figure 4, Friedmann, *p* = 0.008) and at the instep area (Figure 5, Friedmann, *p* = 0.015) differed significantly between conditions of partial body weight, transfer, and full body weight. No changes in the local maximum compression forces at the malleolus lateralis (Figure 6, Friedmann, *p* = 0.245) could be measured between partial body weight, transfer, and full body weight conditions. The lowest maximum compression force was observed for partial body weight loading (0.33 kPa [IQR 0.29–1.13 kPa] for the heel and for the lateral instep [0.24 kPa (IQR 0.22–0.38)]. The highest maximum compression force was 27.3 kPa [IQR 17.5–27.6 kPa] for the heel and 2.10 kPa [IQR 0.98–2.25] for the lateral instep.

### 3.3 Comparison of global and local compression forces at pressure injury-prone body sites

**Figure 7:**
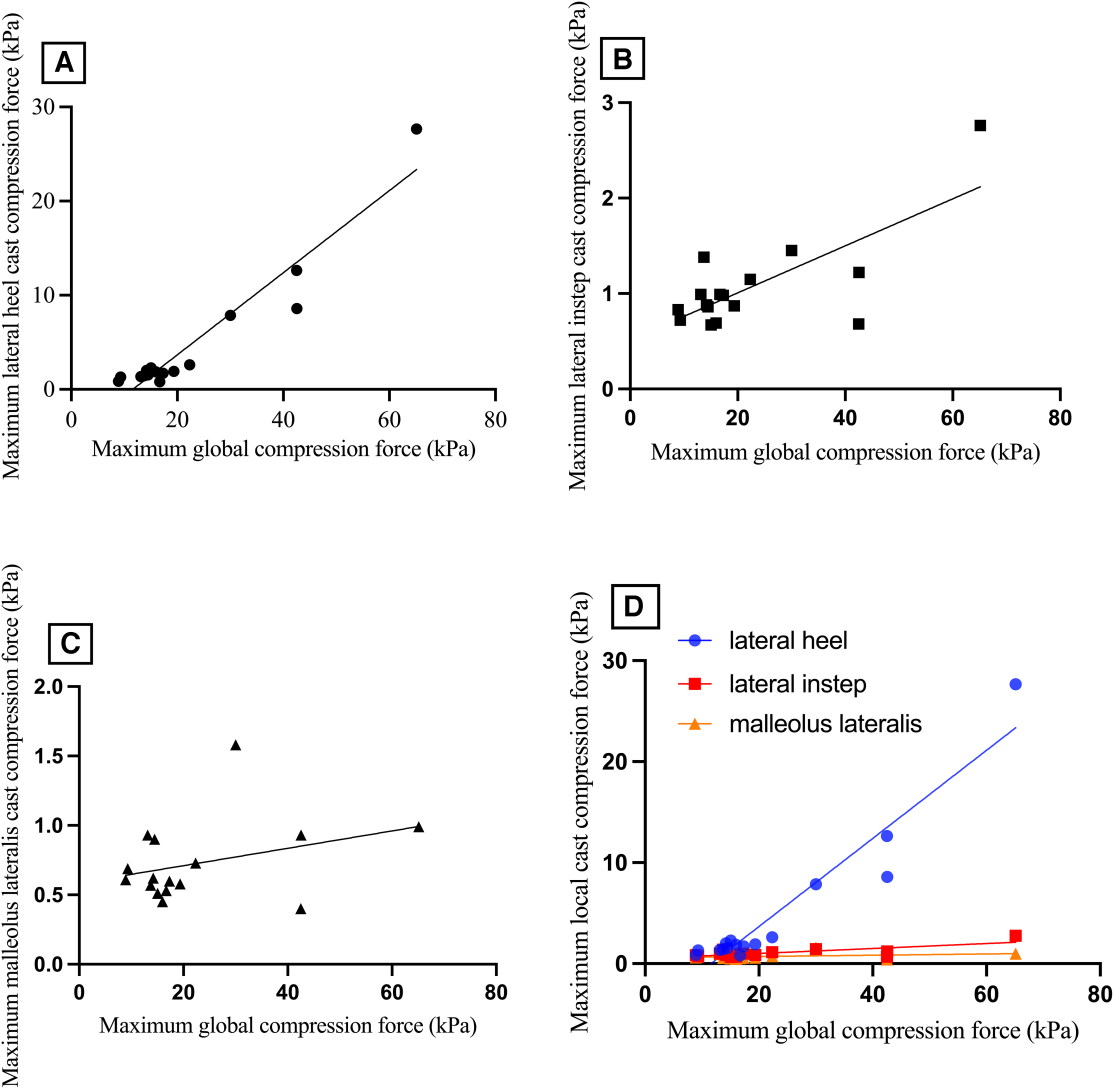
Maximum compression forces observed (**A**) at the lateral heel area, (**B**) the lateral instep area, and (**C**) the malleolus lateralis compared to global maximum compression forces during the three body postures (partial/ full body weight loading/ transfer). (**D**) comparison of maximum local cast compression force (kPa) at the body sites prone to pressure injuries (the lateral heel, the lateral instep, and the malleolus lateralis) with maximum global compression force. kPa, kilopascal.

The global maximum compression forces inside the short leg cast were compared to the maximum local compression forces measured at body sites prone to PI development (the heel, the lateral instep, and malleolus lateralis) during the three body postures (partial/ full body weight loading, and transfer). The global and local maximum compression forces revealed a high correlation for the lateral heel (*ρ* = 0.82, *p* < 0.001) and a moderate correlation at the lateral instep area (*ρ* = 0.51, p = 0.04, Figure 7). We found a low correlation between the global compression forces and the local maximum compression forces at the malleolus lateralis showing a trend towards a statistical significance (*ρ* = 0.448, *p* = 0.055).

### 3.4 Correlation between local compression forces and changes in skin physiology parameters

Regarding skin physiology parameters, data was collected at the lateral instep before the application and immediately after removing the short leg cast. The lateral instep was chosen because the local maximum compression force correlated statistically significantly with the global maximum compression force (Figure 7B).

**Figure 8:**
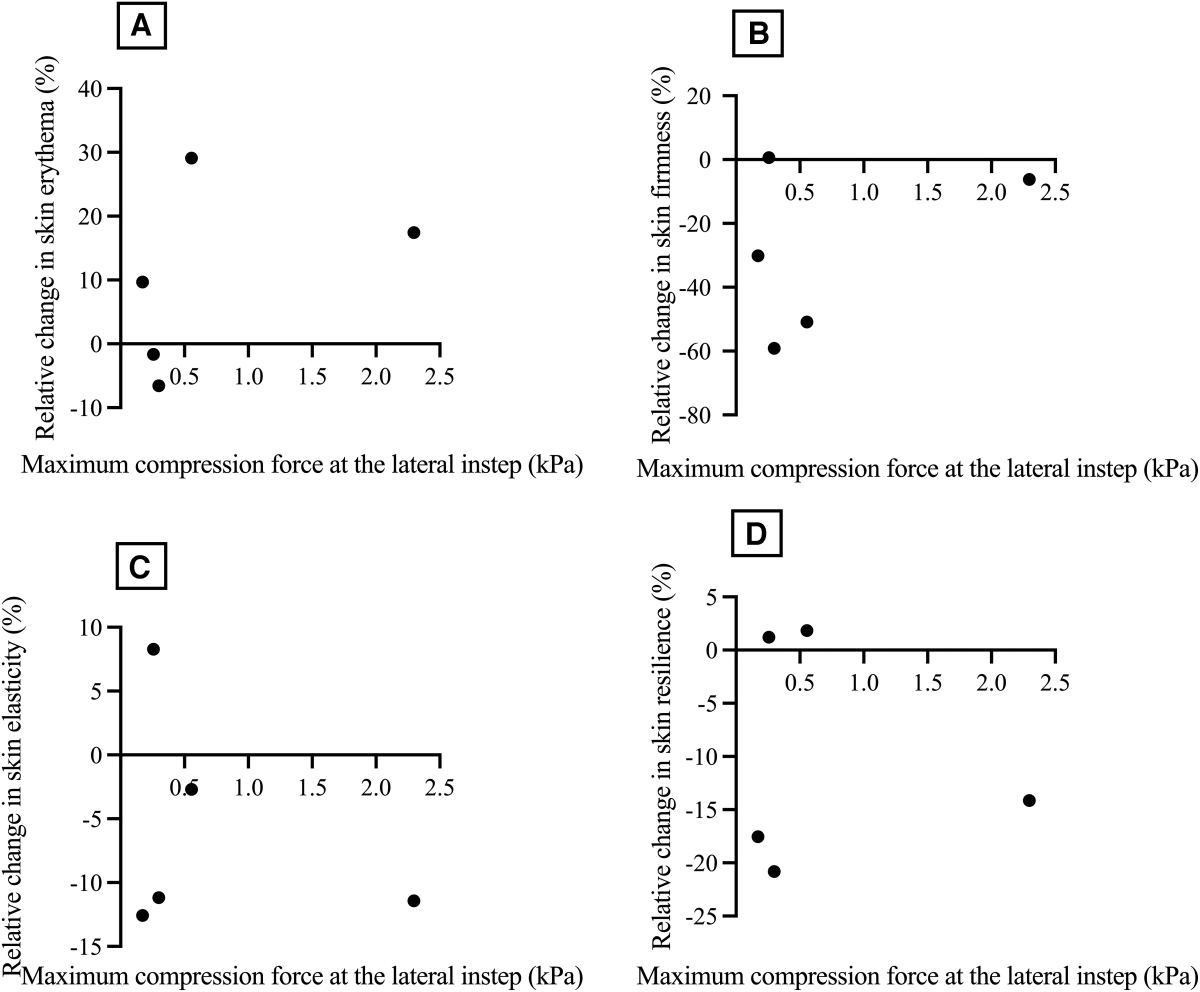
Relative change in skin erythema (%) (**A**), relative change in skin firmness (%) (**B**), relative change in skin elasticity (%) (**C**), and relative change in skin resilience (%) (**D**), related to maximum compression force at the lateral instep (kPa). kPa, kilopascal.

Relative changes in erythema development (Figure 8A) and in skin firmness (Figure 8B), skin elasticity (Figure 8C), and skin resilience (Figure 8D) were calculated and plotted against the maximum compression forces measured at the lateral instep. Neither the relative change in skin erythema (Figure 8A, Spearman *ρ* = 0.5, *p* = 0.391), skin firmness (Figure 8B, Spearman *ρ* = −0.1, *p* = 0.873), skin elasticity (Figure 8C, Spearman *ρ* = 0.1, *p* = 0.873) nor skin resilience (Figure 8D, Spearman *ρ* = −0.3, *p* = 0.624) revealed a statistically significant correlation with maximum compression force at the lateral instep.

## 4 Discussion

This study provides first insights into the potential benefit of incorporating pressure sensors into casts to continuously monitor skin exposure and assess the risk of developing PIs. Many casts have been designed to immobilize the lower limb and reduce the pressure load, enabling less load bearing. A total contact cast is considered to be the best approach to reduce plantar loading in patients with neuropathic PIs (37–39), while the current gold standard for noninvasive fracture immobilization allowing weight-bearing is the patella tendon-bearing cast (40–43).

### 4.1 Global maximum compression forces during predefined body postures

Our study measured the global maximum compression force within a short leg cast across various body positions with increased loading. To our knowledge, no previous attempts have been made to conduct experiments involving variable loading within a short leg cast equipped with pressure sensors in a pediatric sample. Our results demonstrated a significant difference in the global maximum compression force between conditions involving partial and full body weight, and during transfer. Notably, we could objectively quantify the increase in compression force inside a short leg cast in a pediatric sample with higher leg loading.

### 4.2 Local maximum compression force during predefined body postures

As PIs typically occur over bony prominences, we further analyzed the impact of different body positions with increased loading on the local compression force over anatomical risk areas.

We could show a significant difference in local compression force at the instep area and heel with different body weight loading. Our findings align with a study investigating the main weight-bearing area within a conventional patella tendon-bearing cast based on a pressure-sensitive insole using the Tekscan F-SCAN system. Under full loading conditions, the plantar region, including the heel area, was identified to be the main weight-bearing area (44).

Furthermore, the anterodistal lower leg was identified as the highest total contact cast wall load during full body weight loading (45), which is congruent to our findings in a short leg cast. In contrast, we could not observe any significant changes in local compression force during different body postures at the malleolus lateralis. We hypothesize that the malleolus lateralis experienced a greater exposure to shear forces than compression force.

A significant factor in developing PIs is localized ischemia due to obstruction or occlusion of blood vessels in soft tissues by external pressure (46–50), potentially caused by the application of casts. The critical threshold for obstructing blood flow and inducing ischemia, which has the potential to lead to the development of PIs, was determined at 16 kPa (51). The heel exhibited the highest compression force, reaching up to 27.3 kPa, thus significantly surpassing the critical threshold for obstructing blood flow and potentially causing ischemia. In the case of the digital augmented total contact cast, the maximum peak pressure reached an even higher level, measuring at 128.5 kPa at the rearfoot during walking (33). In a digitally augmented short leg cast using Tekscan FlexiForce A201 sensors, the mean peak force at the plantar surface of the calcaneal tuberosity was measured at 31.1N during full loading (52).

### 4.3 Comparison of global and local compression forces at pressure injury-prone body sites

Additionally, we investigated the association between global compression forces and local compression forces measured at anatomical areas prone to PI development across three predefined body postures with full or partial body weight loading and the transfer in between. Our findings revealed a robust and statistically significant correlation between the global and local maximum compression forces at the lateral heel and the lateral instep area. This finding is particularly significant because measuring the global compression force within a short leg cast is technically more straightforward than measuring the local compression force over anatomical areas susceptible to PIs. It can be challenging to accurately position pressure sensors within a cast, specifically over the relevant anatomical area, as they are susceptible to displacement. Placing the pressure sensors directly onto the skin may present issues, as the sensors themselves can contribute to developing PIs.

If the pressure inside the short leg cast reaches a critical level, a potential increase in the local compression force, posing a risk of causing ischemia, can be assumed. It is essential to report that the healthy children involved in this study did not experience any pain or discomfort, even during high pressure that obstructed blood flow. This could indicate that children may be unable to perceive or recognize critically high compression values. On the other hand, the cast was worn for approximately one hour, which may not have been sufficient to cause significant discomfort. Nevertheless, identifying the potential risk of developing PIs would be beneficial to take early preventive actions and avoid any potential side effects. Finally, comparing pressure measurements with other studies is difficult, as they all report different units of measurement, cast techniques, and digital sensors.

### 4.4 Correlation between local compression forces and changes in skin physiology parameters

Multiple studies have demonstrated that relying solely on pressure measurements at the skin surface is inadequate for preventing PIs, particularly when the PI occurs deep in the tissue, causing subcutaneous damage beneath intact skin (53–55). The skin microclimate, encompassing factors such as temperature, humidity, compression, and shear forces, significantly promotes the development of PIs (56). Changes in the functional skin barrier occur before any visible alterations become clinically apparent (57). Prolonged loading (58) and reduced air convection contribute to the local accumulation of heat and humidity (59, 60). Unloading triggers hyperemia, leading to an elevation in skin temperature (61). Elevated skin temperature can weaken the cohesive strength of the stratum corneum (62) and compromise skin integrity (60).

Therefore, we compared how local compression forces affect changes in skin physiology parameters, including erythema development, skin firmness, skin elasticity, and skin resilience. The data was collected at the lateral instep before applying the short leg cast and immediately after removal. We selected the lateral instep because there was a statistically significant correlation between the local maximum compression force and the global maximum compression force. However, no statistically significant correlation was found between the relative change in skin erythema, skin firmness, skin elasticity, or skin resilience and the maximum compression force at the lateral instep. We suspect this lack of correlation may be attributed to the short time spent in the predefined body position and the overall brief period in which the short leg cast was worn. In a study by Kottner et al., increased erythema indices were observed immediately after loading and 15 min after offloading in a population of healthy adults immobilized in a supine position for 90 and 150 min (58). PIs in subdermal tissues beneath bony prominence typically occur between the first hour and 4 to 6 h after loading in a supine position. Additionally, they proposed that PIs occur sooner in a sitting position due to the greater loading compared to a supine position (63).

It is important to emphasize that our study was a short-term investigation and does not reflect the duration of cast wear in clinical practice, where children are often immobilized in casts for weeks. Therefore, we consider the maximum duration of 104 min cast wearing a limitation.

Additional studies involving longer periods of immobilization and additional sensors to measure compression, shear forces, temperature, and humidity could clarify the risk factors associated with critical conditions. To achieve this, it would be desirable to have a multi-parameter sensing system that can be directly integrated into the cast without compromising its stabilizing properties or increasing the risk of developing PI.

In conclusion, integrating sensors into casts holds great potential for continuously monitoring skin exposure and early recognition of critical conditions in developing PIs. As a result, by combining the accumulation of digital information with individual skin characteristics, it may be possible to create a model that can predict the individual risk of developing PIs.

## 5 Limitations of the study

Regarding the methodological limitations of this preliminary study, firstly, the sample size of 5 participants was small, reducing the statistical power. In addition, the limited wearing, and the recording of only a few risk factors do not allow a holistic evaluation of the risk of developing pressure injuries while wearing a cast, which was not the aim of the current study. Secondly, the validity and reliability of Tekscan sensors for pressure measurements on soft tissue and bent surfaces can be challenging (64, 65). The measurement errors with Tekscan sensors can be as high as 34%, even for measurements conducted on flat surfaces (65). According to Wettenschwiler et al., little is known about the validity and reliability of measurements on the skin of human participants, which is much more complex regarding temperature, humidity and surface curvature (66). For the evaluation of local compression pressures, the same sensels were considered from the pressure mapping sensor for each participant, assuming a similar and accurate sensor placement. However, this was difficult to control as the sensor placement was slightly affected when applying the cast and further displaced when removing the cast. In addition, the interpersonal variability in body size and body shape could have added some measurement uncertainties. To address these limitations, we suggest further studies involving a larger sample size and repeated intra-individual multi-parameter measurements over a more extended period of cast wearing. This is crucial for a more faithful representation of the clinical environment where pediatric patients wear a cast over several weeks.

## Data Availability

The raw data supporting the conclusions of this article will be made available by the authors, without undue reservation.
